# Considerations for establishing occupational exposure limits for small molecule kinase inhibitors in drug development

**DOI:** 10.3389/ftox.2026.1743558

**Published:** 2026-03-26

**Authors:** Tyler Nicholas, Eric Morinello, Chandrika Moudgal, Alexandre Beaulieu, Jessica Graham

**Affiliations:** Genentech, Inc., Development Sciences, Translational Safety, South San Francisco, CA, United States

**Keywords:** health hazard data, health-based exposure limit, occupational exposure band, occupational exposure limit, risk assessment, small molecule kinase inhibitor

## Abstract

**Introduction:**

Protein kinases regulate various cell cycle functions, and small molecule kinase inhibitors (SMKIs) represent a growing class of therapeutics. In the pharmaceutical industry, occupational toxicologists enable research and development (R&D) and manufacturing activities for SMKIs by deriving health-based exposure limits (HBELs), including occupational exposure limits (OELs) and occupational exposure bands (OEBs), often in data-limited R&D settings.

**Methods:**

The objective of this study was to evaluate health hazard data for FDA-approved SMKIs and apply a quantitative framework to estimate OELs and map them to OEBs to guide occupational health and safety practices. We compiled health hazard data for SMKIs (n = 86) from literature sources and drug labels including data on genotoxicity, carcinogenicity, developmental and reproductive toxicity (DART), minimum daily therapeutic dose, and sensitive subpopulations. For orally administered SMKIs (n = 83), we estimated OELs using a standardized dose-based approach that incorporates adjustment factors and bioavailability. Estimated OELs were then mapped to OEBs and evaluated by target family.

**Results:**

Estimated OELs ranged from 0.05 to 96 µg/m^3^, with 82% falling between 1 and <100 µg/^3^, spanning OEB 2 and OEB 3A. Developmental toxicity was regarded a class effect, while genotoxicity and carcinogenicity were less frequent and varied by target family.

**Discussion:**

Several target families, including JAK, FGFR, MEK, mTOR, PI3K, and VEGFR inhibitors, exhibited lower estimated OELs and may warrant more protective OEBs in data-limited R&D settings. This analysis demonstrates that a default OEB of 1 to <10 µg/m^3^ (3A) is likely protective for most SMKIs. These findings provide a quantitative, mechanism-informed framework to guide occupational risk management for SMKIs.

## 1 Introduction

Protein kinases are a family of enzymes that catalyze protein phosphorylation. Phosphorylation is regulated by protein kinases and plays a critical role in cell growth, differentiation and development (Cicenas et al., 2018). Kinase activity is often dysregulated in many neurological, immunological, and cardiovascular diseases, making protein kinases frequent therapeutic targets. As of 31 March 2025, the United States Food and Drug Administration (FDA) has approved 86 small molecule kinase inhibitors (SMKIs), and there are approximately 180 SMKIs in clinical trials worldwide with many more in development (Roskoski, 2025). Clinical indications for SMKIs span multiple therapeutic areas, including inflammatory diseases, cancers (solid and non-solid tumors), and cancer-associated immunological states (e.g., chemotherapy-induced myelosuppression) (Roskoski, 2025).

Since protein kinases are involved in many cellular processes and disease areas, SMKIs have become commonplace in pharmaceutical research and development (R&D) pipelines. Since the first SMKI was approved by the FDA in 2001, SMKIs have evolved in terms of potency and pharmacological mechanism of action (MOA) by gaining the ability to target multiple protein kinases (polypharmacy) and conformational states (e.g., imatinib and dasatinib) (Coh et al., 2021). A conformational state refers to the different physical shapes the molecule can take, which determines whether it is active or inactive and thus able to carry out its function of adding phosphate groups to other proteins to activate them. FDA-approved SMKIs currently target a small fraction (∼25) of the 518 members of the protein kinase family, about half of which (244) are hypothesized to be involved in disease pathogenesis (Manning et al., 2002), which presents a significant opportunity to address potentially unmet therapeutic needs.

Due to their growing presence in pharmaceutical development and manufacturing, occupational toxicologists are frequently tasked with assessing molecules in the SMKI therapeutic class for potential health hazards to support worker safety. To do this, occupational toxicologists establish health-based exposure limits (HBELs), such as occupational exposure limits (OELs) which reflect airborne concentrations that are anticipated to be protective in the workplace (Naumann and Sargent, 1997; Sargent and Kirk, 1988). A robust assessment and derivation process is utilized to develop OELs, which are generally established when sufficient nonclinical and clinical data is available (i.e., Phase 3 clinical trials). These limits are utilized to guide handling practices (e.g., personal protective equipment) and manufacturing equipment selection (e.g., isolators) as part of a performance-based approach (Naumann, 2006; International Society for Pharmaceutical Engineering, 2017). In addition to the limits conveying the severity of their pharmacological and/or toxicological properties, the assessments (i.e., monographs) provide a level of understanding of the human health hazards (e.g., reproductive toxicant) associated with the molecule at hand. However, in early development, although exposure limits are still needed to guide handling practices, limited data availability for active pharmaceutical ingredients (APIs) poses a challenge for occupational toxicologists and related safety experts responsible for establishing refined OELs. To assist with this challenge, hazard banding strategies such as occupational exposure bands (OEBs) are established based on historical experience and any relevant literature (Naumann, 2006; Deveau et al., 2015). The OEB is the airborne concentration range where the speculative OEL is anticipated to fall once additional data is available. Because APIs such as SMKIs can vary significantly in potency, a cautious approach is generally adopted when establishing the initial OEB. This strategy aims to ensure that the initial limit is protective, thereby minimizing the risk of future reductions (i.e., the necessity for changes in manufacturing sites, equipment, and controls) and preventing concerns about possible over-exposures.

**FIGURE 1:**
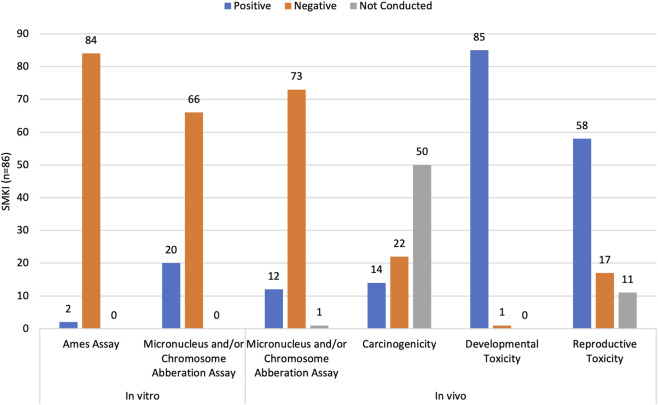
Assessment pathway for mapping OEBs to FDA-approved SMKIs. Available nonclinical and clinical data for SMKIs are used for hazard identification, followed by selection of a conservative POD based on the minimum daily therapeutic dose. Adjustment factors are applied to address uncertainty and estimate an OEL. The OEL is then mapped to an OEB to support handling and risk management decisions in data-limited R&D settings.

To address this emerging need, we compiled and summarized available health-hazard data for FDA-approved SMKIs. This article reviews class-specific health hazards and potential differences by kinase targets or families. Using complete data, we characterized primary hazards and estimated OELs; these insights then informed guidance for mapping OEBs for SMKIs in data-limited R&D settings. Several banding systems relevant to these molecules have been described in the literature (Naumann, 2006; Ader et al., 2005; Graham et al., 2020); they propose speculative OEL ranges (µg/m^3^) to guide synthesis, handling, and manufacturing when a molecule-specific OEL cannot yet be defined (Naumann and Weideman, 1995). Given the importance of initial OEB assignment, we used the available hazard information to propose a default OEB for SMKIs in data-limited R&D settings and to identify kinase families that may warrant more protective OEBs based on MOA. The OELs and OEBs estimated from this broad approach are not intended to substitute for company-specific limits, which are generally based on proprietary toxicology data. Instead, they provide an assessment pathway to guide estimation of OELs and mapping OEBs for early development molecules within this therapeutic class, as shown in Figure 1.

## 2 Materials and methods

### 2.1 Identification and compilation of FDA-approved SMKIs

We obtained a list of FDA-approved SMKIs from Roskoski 2025 and the FDA approved drug database (Roskoski, 2025; Drugs@FDA). Inclusion criteria comprised FDA-approved SMKIs approved as of 31 March 2025, while exclusion criteria included non-small molecule therapeutics and products that were not classified as kinase inhibitors. This resulted in a total of 86 SMKIs for evaluation. We acquired the primary therapeutic targets as well as nonclinical and clinical data for each marketed product from Roskoski 2025 and the respective drug label (prescribing information). We also obtained the drug labels from the from the FDA website (Drugs@FDA). The drug label data was reviewed and extracted by at least two authors, with discrepancies resolved through consensus.

### 2.2 Evaluation of nonclinical data for FDA-approved SMKIs

We complied nonclinical data consisting of genotoxicity (mutagenicity, clastogenicity, and aneugenicity), carcinogenicity, as well as developmental and reproductive toxicity (DART) information for all SMKIs based on the information presented in each drug’s label. We utilized criteria outlined in the United Nation’s Globally Harmonized System for Classification and Labeling (UN GHS) as a guide to determine whether an SMKI was “positive” for carcinogenicity, developmental toxicity, and/or reproductive toxicity (United Nations Economic Commission for Europe, 2023). We regarded a molecule as positive for developmental toxicity or reproductive toxicity where relevant study data was available, and the results would warrant classification as a developmental or reproductive toxicant. In cases where findings were equivocal or borderline, a conservative classification was applied if DART effects were observed at or near therapeutically relevant doses. Ambiguous cases (e.g., embryo-fetal findings were secondary to maternal toxicity) were reviewed by at least two authors and resolved through consensus. Similarly, we considered a molecule as positive for carcinogenicity where relevant study data was available, and the results would warrant classification for carcinogenicity. Finally, we deemed a molecule as positive for aneugenicity or clastogenicity if they were reported as being positive for aneugenicity or clastogenicity or acting *via* an aneugenic or clastogenic mechanism (e.g., positive in a micronucleus assay *via* an aneugenic or clastogenic mechanism).

### 2.3 Evaluation of clinical data for FDA-approved SMKIs

We collected clinical data on the intended route(s) of administration, minimum daily therapeutic dose, and information on sensitive subpopulations. For instances where multiple therapeutic dose levels were specified, we recorded the lowest recommended therapeutic dose (mg/day). We made considerations for inclusion of data on sensitive subpopulations (e.g., populations that may experience potential higher systemic exposures) based on whether reduced dosing was recommended for renal and/or hepatic impairment in addition to whether a drug is anticipated to have potential interactions with CYP inducers and/or inhibitors.

### 2.4 Estimation of OELs and mapping of OEBs for FDA-approved SMKIs

To understand appropriate OEBs (exposure range for data-limited molecules) for SMKIs, we estimated OELs for FDA-approved SMKIs intended to be administered *via* the oral route (*n* = 83). We excluded SMKIs administered *via* other routes from the OEB analysis including netarsudil (RHOPRESSA®) (ocular), temsirolimus (TORISEL®) (IV), and trilaciclib (COSELA®) (IV).

The OELs (and subsequent OEBs) estimated from this broad approach are not intended to substitute for the respective company-established limits which are generally based on proprietary toxicology data as well as a more detailed review of available information. The estimated OELs and mapped OEBs herein utilize a generalized approach (as detailed below) with the intention of providing guidance in the establishment of speculative HBELs for data-poor SMKIs.

#### 2.4.1 Estimation of OELs

For this assessment, we estimated OELs for SMKIs utilizing the therapeutic dose divided by the safety factor also known as the “fraction of the minimum therapeutic dose” approach as described in Faria et al. (2016), Ku (2000). We utilized a general OEL derivation equation (Equation 1) as described in the literature (Naumann and Sargent, 1997; Faria et al., 2016; Sargent et al., 2013; Sussman et al., 2016). To estimate each OEL, we divided the minimum daily therapeutic dose or point of departure (POD), which was used as a surrogate lowest observed adverse effect level (LOAEL) for the critical effect, by adjustment factors in Equation 1, which are further described below. Application of these factors results in the information conveyed in Equation 2. This “margin of safety” approach is commonly used in standard pharmaceutical OEL derivation practice as pharmacology is often the most sensitive effect of an API (Faria et al., 2016; Ku, 2000; Lehman, 1954).

Equation 1. General OEL derivation equation

$$OEL\;\left(\frac{\mu g}{m^{3}}\right)=\frac{POD\;for\;critical\;effect\;\left(\mu g\right)\times BA}{F1\times F2\times F3\times F4\times F5\times V\;\left(m^{3}\right)}$$

Equation 2. General OEL derivation equation with SMKI assumptions

$$SMKI\;OEL\;\left(\frac{\mu g}{m^{3}}\right)=\frac{minimum\;daily\;therapeutic\;dose\;\left(\mu g\right)\times 50\%}{1\times 10\times 1\times \left(5\;or\;10\right)\times 10\times \left(10\;m^{3}\right)}$$

##### 2.4.1.1 POD - point of departure (LOAEL or NOAEL) for the critical effect

In order to estimate the OELs for each SMKI, we determined the critical effect and applied adjustment factors (Henschler, 1991). Pharmacology is often the driving factor (critical effect) when establishing OELs for APIs as pharmacology is often observed before toxicity (e.g., therapeutic index >1) (Faria et al., 2016; Ku, 2000). Therefore, we utilized the minimum daily therapeutic dose (LOAEL) as the POD. In many cases for SMKIs, there are reduced dosing recommendations when adverse effects are observed at the recommended therapeutic dose. It is important to note that the minimum daily therapeutic dose utilized for the purposes of this OEL estimation, is the lowest dose recommended after such dosage adjustments downward. Therefore, the minimum daily therapeutic dose utilized is not synonymous with the recommended daily therapeutic dose.

##### 2.4.1.2 F1 - Interspecies adjustment factor

Since we utilized human data, this factor is applied to account for differences between animals and humans (i.e., POD from an animal study) and set to 1 across SMKIs.

##### 2.4.1.3 F2 - Interindividual variability

When estimating OELs for APIs, we assumed the potentially exposed population is healthy workers of both sexes, all races, and ranging in age from 18 to 65 years (Sargent et al., 2013). It is generally recommended to use a default factor of 10 to account for interindividual variability for the worker population (International Society for Pharmaceutical Engineering, 2017; Dourson and Stara, 1983; Calabrese, 1985; Hatti et al., 1987; International Council for Harmonisation of Technical Requirements for Pharmaceuticals for Human Use, 2021). Notably, the use of a factor of 10 is anticipated to be conservative (Naumann and Sargent, 1997). Other values have also been recommended, for example the European Chemicals Agency (ECHA) recommends a factor of 5 to account for interindividual variability for workers (European Centre for Ecotoxicology and Toxicology of Chemicals, 2010; European Chemicals Agency, 2012). In this instance, we applied a higher factor of 10 across SMKIs.

##### 2.4.1.4 F3 - Exposure duration adjustment factor

Given the POD is the minimum daily therapeutic dose and steady state is assumed to be reached, we applied a factor of one across SMKIs (Sargent et al., 2013). We do not anticipate an additional adjustment to be necessary given the doses are daily and the POD is established from studies where steady state exposure is achieved (Sargent et al., 2013).

##### 2.4.1.5 F4 - Severity of effect

We evaluated SMKIs for their potential to cause genetic toxicity, carcinogenicity, developmental toxicity (i.e., teratogenicity) and reproductive effects in males or females (Supplementary Table S1). We applied a factor of 5 when developmental and/or reproductive toxicity was observed in nonclinical studies per ICH guidance, and if this effect was attributed to on-target pharmacology per the drug’s prescribing information (International Council for Harmonisation of Technical Requirements for Pharmaceuticals for Human Use, 2021). We increased this factor to 10 when concerns for genotoxicity and carcinogenicity were present (positive in a carcinogenicity study or positive in an *in vivo* genotoxicity assessment in the absence of a carcinogenicity study) (International Council for Harmonisation of Technical Requirements for Pharmaceuticals for Human Use, 2021). While application of the F4 severity factor may introduce conceptual overlap with the LOAEL-to-NOAEL extrapolation factor (F5), F4 addresses uncertainty related to the type and severity of the observed effect, whereas F5 addresses uncertainty related to estimating a no-effect level from a pharmacologically active dose. Utilizing both factors is consistent with standard pharmaceutical OEL derivation practice and is intentionally conservative for data-limited R&D contexts.

##### 2.4.1.6 F5 - LOAEL-to-NOAEL extrapolation

Typically, a factor of 3–10 is recommended to extrapolate from a LOAEL to a no observed adverse effect level (NOAEL) (Sussman et al., 2016). Since the POD is the minimum therapeutic dose, e.g., a pharmacologically active dose, it is possible that pharmacology could occur 3-fold below this dose, and therefore we selected the higher default factor of 10. The use of a factor of 10 in extrapolating from a minimum therapeutic dose to a no-effect level is also further supported and described in Ku (2000). Additionally, a cumulative adjustment factor of 1,000 has often been applied for APIs (Dankovic et al., 2015), and holistically, the total adjustment factor applied herein ranges from 1,000 to 2,000 across SMKIs.

##### 2.4.1.7 BA- Bioavailability

Adjusting for differences in BA can have a significant effect on OELs. Historically, for orally administered small molecules, inhalation BA (deposition and absorption in the respiratory tract) is conservatively assumed to be complete at 100% (Naumann and Sargent, 1997). SMKIs are anticipated to have high oral BA with a portion of the inhaled dose being ingested (i.e., *via* mucociliary transport from the lung). Oral BA values were available for approximately half (47%; 40/86) of FDA-approved SMKIs, averaging at 59.8%. However, due to uncertainty with regards to the average oral BA of this therapeutic class, we assumed a default of 50% absorption *via* the oral route per ECHA guidance (European Chemicals Agency, 2012).

##### 2.4.1.8 V - Breathing rate

We applied a breathing rate of 10 m^3^/8-h workday (Ku, 2000; European Chemicals Agency, 2012).

##### 2.4.1.9 Bringing it all together

Applying these factors results in the information conveyed in Equation 2. While we applied each factor independently, they are not necessarily independent of one another and their interdependence is an important consideration (Sussman et al., 2016). For example, developmental and reproductive toxicity are generally known (pharmacological) effects of SMKIs, and it is anticipated that in estimating a limit anticipated to not be pharmacologically active, these health hazards would also be accounted for. The factor of F5 is intended to extrapolate from the POD of a NOAEL and cover concerns due to pharmacology. Additionally, F4 is intended to account for the severity of the effect and applied where there are concerns for genotoxicity, carcinogenicity, developmental and/or reproductive toxicity. The application of a factor for F4 in the case of SMKIs, which pose DART concerns, may be considered “double-dipping”, but use of F4 (i.e., F4 = 5) is a common practice in the industry (as a conservative measure to account for uncertainty) where DART is anticipated based on the MOA and not an off-target effect (Dankovic et al., 2015; Gould et al., 2016).

#### 2.4.2 Mapping of OEBs

Banding systems are not harmonized across the industry and thus differ slightly from company to company. The Roche/Genentech banding system aligns with internal validated control paradigms, and we mapped estimated OELs for individual SMKIs to OEBs according to this system’s predefined airborne concentration ranges (Table 2; Figure 4).

##### 2.4.2.1 OEB 1

OEB 1 reflects an airborne concentration range of ≥100 μg/m^3^, indicating a low hazard or non-hazardous molecule for which a laboratory or production facility with limited containment will generally be acceptable.

##### 2.4.2.2 OEB 2

OEB 2 reflects an airborne concentration range of 10 to <100 μg/m^3^, indicating a moderately hazardous molecule for which a laboratory or production facility with standard but not highly sophisticated containment will generally be acceptable.

##### 2.4.2.3 OEB 3A and 3B

OEB 3 is split into 3A and 3B bands and reflects airborne concentration ranges of 1 to <10 μg/m^3^ (3A) and 0.05 to <1 μg/m^3^ (3B), respectively, both of which indicating high hazard molecules for which a modern laboratory and production facility with excellent levels of containment are needed—well-trained staff is generally required.

##### 2.4.2.4 OEB 4

OEB 4 reflects an airborne concentration range of ≤0.05 μg/m^3^, indicating a very high hazard molecules of extreme potency/toxicity for which purpose-built laboratories and production conditions must be defined—highly trained and experienced staff is required.

## 3 Results

### 3.1 Summary of health hazard data for FDA-approved SMKIs

**FIGURE 2:**
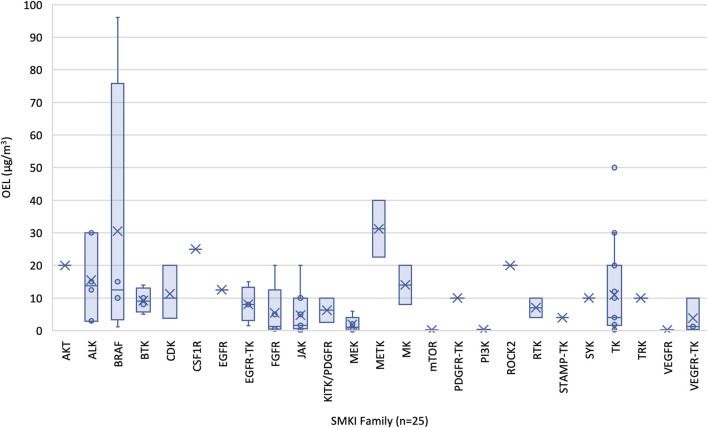
Summary of health hazards of FDA-approved SMKIs. An evaluation of the nonclinical toxicity studies for 86 SMKIs. The nonclinical data was evaluated with a focus on genotoxicity (mutagenicity, clastogenicity, aneugenicity), carcinogenicity, and developmental and reproductive toxicity (DART) as these endpoints are considered relevant to occupational risk assessment.

A total of 86 SMKIs have been approved by the FDA as of 31 March 2025, 86% of which (74/86) are marketed for oncology indications with the majority (97%; 83/86) being administered orally. Clinical and nonclinical data available for each FDA-approved SMKI was evaluated from the prescribing information and is summarized in Figure 2 and Table 1, and Supplementary Table S1. Across FDA-approved SMKIs, reported toxicities included cardiotoxicity, hematotoxicity, hepatotoxicity, as well as DART. These endpoints were classified as “not available” when data were absent, and missing data did not default to positive classification without supporting information. The target(s) for each SMKI assessed are presented in Supplementary Table S1, with some molecules having a primary target and secondary targets, or several primary targets.

**TABLE 1:** Summary of health hazards of FDA-approved SMKIs. Overview of health hazards associated with SMKIs organized by target family.

Target family	*N* =	Aneugenicity			Micronucleus *(in vitro)*			Micronucleus *(in vivo)*			Carcinogenicity			Developmental toxicity			Reproductive toxicity		
		+	−	N/A	+	−	N/A	+	−	N/A	+	−	N/A	+	−	N/A	+	−	N/A
AKT	1	1	−	−	−	1	−	1	−	−	−	−	1	1	−	−	1	−	−
ALK	6	3	−	3	4	2	−	4	2	−	−	−	6	6	−	−	4	−	2
BRAF	4	−	−	4	−	4	−	−	4	−	−	−	4	4	−	−	3	−	1
BTK	4	1	−	3	−	4	−	−	4	−	−	1	3	4	−	−	−	4	−
CDK	4	1	−	3	2	2	−	1	2	1	1	2	1	4	−	−	4	−	−
CSF1R	1	−	−	1	−	1	−	−	1	−	−	1	−	1	−	−	1	−	−
EGFR	1	−	−	1	−	1	−	−	1	−	1	−	−	1	−	−	1	−	−
EGFR-TK	4	−	1	3	−	4	−	−	4	−	−	1	3	4	−	−	3	1	−
FGFR	5	−	−	5	1	4	−	−	5	−	−	1	4	5	−	−	2	1	2
JAK	11	−	2	9	4	7	−	−	11	−	2	9	−	10	1	−	7	4	−
KITK/PDGFR	2	−	−	2	1	1	−	−	2	−	−	−	2	2	−	−	2	−	−
MEK	5	1	−	4	−	5	−	2	3	−	−	2	3	5	−	−	3	1	1
METK	2	−	−	2	−	2	−	−	2	−	−	−	2	2	−	−	−	−	2
MK	2	−	−	2	1	1	−	−	2	−	−	−	2	2	−	−	2	−	−
mTOR	3	−	−	3	−	3	−	−	3	−	1	1	1	3	−	−	3	−	−
PDGFR-TK	1	−	−	1	1	−	−	−	1	−	−	−	1	1	−	−	1	−	−
PI3K	1	−	−	1	1	−	−	−	1	−	−	−	1	1	−	−	1	−	−
ROCK	2	−	−	2	−	2	−	−	2	−	−	−	2	2	−	−	1	−	1
RTK	2	−	−	2	−	2	−	1	1	−	−	−	2	2	−	−	2	−	−
STAMP-TK	1	−	−	1	−	1	−	−	1	−	−	−	1	1	−	−	1	−	−
SYK	1	−	−	1	−	1	−	−	1	−	−	1	−	1	−	−	1	−	−
TK	18	2	−	16	4	14	−	2	16	−	7	3	8	18	−	−	11	5	2
TRK	1	−	−	1	−	1	−	−	1	−	−	−	1	1	−	−	1	−	−
VEGFR	1	−	−	1	−	1	−	1	−	−	−	−	1	1	−	−	1	−	−
VEGFR-TK	3	−	−	3	−	3	−	−	3	−	2	−	1	3	−	−	2	1	−

#### 3.1.1 Genotoxicity and carcinogenicity

Among the FDA-approved SMKIs, genotoxicity data was available for all molecules. Most SMKIs were negative in Ames assays (98%; 84/86) as well as micronucleus assays and chromosome aberrations assays *in vitro* (77%; 66/86) and *in vivo* (86%; 73/86). These SMKIs have demonstrated a potential to cause chromosomal damage *via* clastogenic or aneugenic mechanisms, consistent with the role of certain protein kinases in chromosomal segregation (Table 1) (Olaharski et al., 2009). Carcinogenicity was assessed for less than half of SMKIs (42%; 36/86) and among these, 39% (14/36) tested positive in nonclinical studies in one or two species, but the clinical relevance of these findings is unknown.

#### 3.1.2 Developmental and reproductive toxicity

Developmental toxicity was observed across nearly all FDA-approved SMKIs (99%; 85/86) suggesting that it could be regarded as a class effect. Reproductive toxicity was reported for most of the molecules (66%; 57/86). Taken together, DART is an anticipated health hazard of concern for this therapeutic class.

#### 3.1.3 Clinical therapeutic dose regimen

**FIGURE 3:**
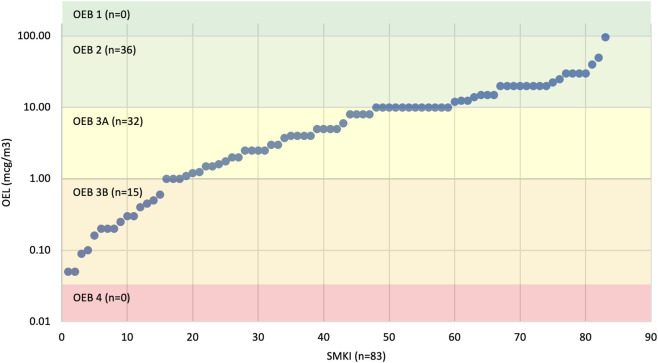
Distribution of estimated OELs for FDA-approved oral SMKIs across OEBs. The estimated OELs for oral SMKIs were mapped to OEBs.

The minimum therapeutic doses (all daily) ranged from 0.5 to 1,000 mg for oral SMKIs (Figure 3). Based on the drug labels, individuals with hepatic and/or renal impairment were frequently reported to be sensitive subpopulations. Of the FDA-approved SMKIs, 52% (45/86) provided dosing modifications or were contraindicated in these subpopulations. Additionally, 85% (73/86) of SMKIs were reported to interact with CYP3A4 inducers and/or inhibitors, with reduced dosing often recommended when administered with CYP3A4 inhibitors.

### 3.2 Estimated OELs and distribution of OEBs across FDA-approved SMKIs

As described in Section 2.4, OELs were estimated for orally administered FDA-approved SMKIs (*n* = 83) using the minimum daily therapeutic dose divided by the safety factor, consistent with approaches described in Faria et al. and Ku (Faria et al., 2016; Ku, 2000). Estimated OELs for individual SMKIs were subsequently mapped to OEBs based on predefined airborne concentration ranges (Table 2).

**TABLE 2:** OEB system. OEBs were mapped to SMKIs based on predefined airborne concentration ranges according to the Roche/Genentech banding system, which aligns with validated control paradigms.

OEB	Airborne concentration range (μg/m^3^)	Hazard description	General handling recommendations
1	≥100	Low hazard or non- hazardous	Molecules for which a laboratory or production facility witd limited containment will be acceptable
2	10 to <100	Moderate hazard	Molecules for which a laboratory or production facility witd standard but not highly sophisticated containment will be acceptable
3A	1 to <10	High hazard	Molecules for which a modern laboratory and production facility with excellent levels of containment are needed. Well-trained staff is required
3B	0.05 to <1		
4	≤0.05	Very high hazard	Molecules of extreme potency/toxicity for which laboratories and production conditions must be defined. Highly trained and experienced staff is required

Across all evaluated SMKIs, estimated OELs ranged from 0.05 μg/m^3^ – 96 μg/m^3^, spanning OEB 2, 3A, and 3B (Figure 3; Supplementary Table S2). Overall, 82% (68/83) of SMKIs had estimated OELs between 1 and <100 μg/m^3^, with 43% (36/83) in OEB 2 (10 to <100 μg/m^3^) and 39% (32/83) in OEB 3A (1 to <10 μg/m^3^). The remaining 18% (15/83) of SMKIs had estimated OELs in OEB 3B (0.05 - <1 μg/m^3^). No estimated OELs exceeded ≥100 μg/m^3^ (OEB 1) or fell below <0.05 μg/m^3^ (OEB 4).

**FIGURE 4:**
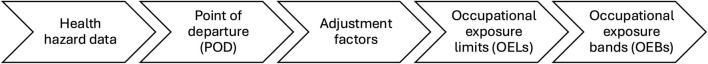
Distribution of estimated OELs for FDA-approved oral SMKIs across target families. An evaluation of health hazard data showed a broad distribution of estimated OELs for oral SMKIs across their respective target families.

Estimated OELs were further evaluated by target family to identify patterns relevant to default OEB assignment for SMKIs in data-limited R&D settings, as shown in Figure 4 and Table 3. For example, several target families had estimated OELs in OEB 3B, including FGFR (2/5), Janus kinase (JAK) (3/11), MEK (2/5), mTOR (2/2), PI3K (1/1), TK (3/18), VEGFR (1/1), and VEGFR-TK inhibitors (1/3). Other target families were largely confined to OEB 2 and OEB 3A.

**TABLE 3:** OEBs for SMKIs organized by target family.

Target Family	*N* =	OEB 1	OEB 2	OEB 3A	OEB 3B	OEB 4
AKT	1	−	1	−	−	−
ALK	6	−	4	2	−	−
BRAF	4	−	3	1	−	−
BTK	4	−	2	2	−	−
CDK	3	−	2	1	−	−
CSF1R	1	−	1	−	−	−
EGFR	1	−	1	−	−	−
EGFR-TK	4	−	1	3	−	−
FGFR	5	−	1	2	2	−
JAK	11	−	3	5	3	−
KITK/PDGFR	2	−	1	1	−	−
MEK	5	−	−	3	2	−
METK	2	−	2	−	−	−
MK	2	−	1	1	−	−
mTOR	2	−	−	−	2	−
PDGFR-TK	1	−	1	−	−	−
PI3K	1	−	−	−	1	−
ROCK	1	−	1	−	−	−
RTK	2	−	1	1	−	−
STAMP-TK	1	−	−	1	−	−
SYK	1	−	1	−	−	−
TK	18	−	7	8	3	−
TRK	1	−	1	−	−	−
VEGFR	1	−	−	−	1	−
VEGFR-TK	3	−	1	1	1	−

### 3.3 Distribution of hazards, OELs, and OEBs across major target families

To further contextualize these family-level distributions, health hazard data were evaluated for major target families with substantial available nonclinical and clinical data. Multiple B-Raf proto-oncogene, serine/threonine kinase (BRAF) inhibitors (*n* = 4), Bruton’s tyrosine kinase (BTK) inhibitors (*n* = 4), cyclin-dependent kinase (CDK) inhibitors (*n* = 4), and JAK inhibitors (*n* = 11), have been approved by the FDA and are currently marketed. Given the number of approved SMKIs, continued interest in pharmaceutical candidates, and availability of health hazard data, these target families were analyzed in greater detail to characterize their health hazard profiles, estimated OELs, and mapped OEBs relevant to occupational safety.

#### 3.3.1 BRAF inhibitors

The RAS/RAF/MEK/ERK mitogen-activated protein kinase (MAPK) pathway plays a key role in the regulation of fundamental cellular processes, including cell growth, development, division, transformation, proliferation, migration, and death (apoptosis). Activating mutations in BRAF kinase, predominantly related to a valine to glutamic acid substitution at residue 600 (V600E), have been observed in approximately 8% of all solid tumors (Pratilas et al., 2012). Such mutations result in constitutive activation of BRAF kinase, which causes dysregulated downstream signaling *via* MEK and ERK, leading to excessive cell proliferation and survival. BRAF mutations are among the most common leading to overactivation of the MAPK pathway, and BRAF-activating mutations are found in at least half of cutaneous melanomas (Cheng et al., 2018).

The BRAF inhibitors dabrafenib (TAFINLAR®), encorafenib (BRAFTOVI®), tovorafenib (OJEMDA®), and vemurafenib (ZELBORAF®) are approved for use as single agents or in combination with other anti-cancer agents in the treatment of patients that meet certain criteria with BRAF^V600^-mutant advanced melanomas or other conditions related to BRAF^V600^ mutation (Novartis Pharmaceuticals Corporation, 2024; Array Biopharma, 2023; Day One Biopharmaceuticals, 2024; Genentech, 2020). All are orally administered, small molecule, ATP-competitive inhibitors that are relatively selective for BRAF^V600^ *versus* wild-type BRAF or other kinases. By inhibiting mutated BRAF, BRAF inhibitors interfere with the MAPK signaling pathway that regulates the proliferation and survival of tumor cells.

##### 3.3.1.1 Overview of key health hazards

Information regarding the adverse effects of BRAF inhibitors is available in the package inserts and health authority approval documentation (Novartis Pharmaceuticals Corporation, 2024; Array Biopharma, 2023; Day One Biopharmaceuticals, 2024; Genentech, 2020). Because of their relative specificity for mutated BRAF, dabrafenib, encorafenib, and vemurafenib were relatively well tolerated in *in vivo* toxicology studies compared to other classes of kinase inhibitors. In each case, tolerability was generally limited by nonspecific toxicities that were not associated with target organs. Notable findings in the nonclinical studies reported with one or more of these products included skin abnormalities, tissue hyperplasia/hyperkeratosis, evidence of hepatotoxicity, and effects on the male reproductive system. Effects on the skin and male reproductive system appeared to be the most sensitive toxicity endpoints in rodents and non-rodents and were generally observed at comparable therapeutic dose levels.

The most common adverse reactions in patients treated with BRAF inhibitors vary by product and indication, but include nonspecific effects such as fatigue, nausea, abdominal pain, and effects on the skin (e.g., rash, dermatitis). Other adverse effects inherent to the MOA include new primary cutaneous or non-cutaneous malignancies, tumor promotion in BRAF wild-type tumors, and skin toxicities. Embryo-fetal toxicity is also typically considered to be a potential class risk. The adverse effects that most commonly result in dose reduction or discontinuation vary among products, patient populations, and concomitant therapies but often include pyrexia, neutropenia, skin toxicities, or squamous cell carcinoma (attributed to a paradoxical effect on MAPK signaling).

##### 3.3.1.2 Mutagenicity and carcinogenicity

None of the approved BRAF inhibitors are known to be mutagenic or clastogenic, and carcinogenicity studies were not conducted (Table 1). *In vitro* experiments have demonstrated that ATP-competitive BRAF inhibitors can paradoxically enhance MAPK signaling in BRAF wild-type cells (Poulikakos et al., 2010). Primary malignancies have been observed in patients treated with BRAF inhibitors are a class risk; however, this occurs *via* a non-genotoxic mechanism related to the MOA and is unlikely to be relevant at far-subtherapeutic exposures relevant to occupational exposure scenarios.

##### 3.3.1.3 Developmental and reproductive toxicity

Based on data from BRAF knockout models and evidence of malformations and/or embryotoxicity in nonclinical models at supra-therapeutic exposures, BRAF inhibitors have been presumed to pose a class risk of embryo-fetal toxicity (Novartis Pharmaceuticals Corporation, 2024; Array Biopharma, 2023; Genentech, 2020). Dedicated studies to evaluate male or female fertility have not been reported (Table 1). Adverse effects on male and female fertility-related endpoints (e.g., hypospermia in males and decreased corpora lutea in females) have been observed in nonclinical toxicity studies at doses resulting in systemic exposures comparable to or greater than those observed in patients at the recommended therapeutic dose (Novartis Pharmaceuticals Corporation, 2024).

##### 3.3.1.4 Sensitive subpopulations

Based on their MOAs, BRAF inhibitors are presumed to pose a class risk of embryo-fetal toxicity as well as additional risks to immunocompromised individuals. The risks described for other potentially sensitive subpopulations are primarily related to molecule-specific metabolic considerations (e.g., hepatic impairment, interactions with CYP inhibitors, *etc*).

##### 3.3.1.5 Other concerns and considerations

None of the marketed BRAF inhibitors have black box warnings. With regards to translational effects, BRAF inhibitors are known to cause embryo-fetal toxicity specifically in rats and rabbits, and this is considered a class effect relevant to safety assessment in humans.

##### 3.3.1.6 Hazard classifications for safety data sheets

Based on health hazard data for the three FDA-approved BRAF inhibitors, GHS classifications for carcinogenicity and reproductive toxicity (developmental as well as male and female fertility) may be warranted for BRAF inhibitors lacking data for these endpoints.

##### 3.3.1.7 Bringing it all together—considerations for estimating OELs for BRAF inhibitors

The MOA and relative specificity for mutant BRAF is anticipated to decrease the risk of effects on healthy workers (as well as exposures due to product carry-over scenarios). BRAF inhibitors, like many kinase inhibitors, have a narrow therapeutic index. Carcinogenicity studies were not conducted for any BRAF inhibitor; however, secondary malignancies have been observed in clinical studies and are considered to be a class risk (Gibney et al., 2013). However, this occurs *via* a non-genotoxic mechanism related to the MOA and is unlikely to be relevant at far-subtherapeutic exposures. Embryofetal development (EFD) effects have been observed in nonclinical studies (Supplementary Table S1) and appear to be limited to supratherapeutic exposures, which is consistent with involvement of wild-type BRAF or other off-target kinase hits; however, these effects have questionable relevance at far-subtherapeutic exposures. While dedicated fertility studies were not reported for the approved BRAF inhibitors, effects were observed in reproductive organs in both males and females. BRAF inhibitors are not exquisitely specific for mutant BRAF and inhibit wild-type BRAF and other kinases at therapeutic doses; however, this lack of specificity is of less concern at far-subtherapeutic exposures. Considering these factors, OELs were estimated for orally administered FDA-approved BRAF inhibitors (*n* = 4) using the therapeutic dose divided by the safety factor approach as described in Section 2.4 and in Faria et al. and Ku (Faria et al., 2016; Ku, 2000). The estimated OELs for BRAF inhibitors range from 1.1 to 96 μg/m^3^ (Figure 4; Supplementary Table S2) spanning OEB 2 and 3A, (Figure 3).

#### 3.3.2 BTK inhibitors

Bruton’s tyrosine kinase (BTK) is a cytoplasmic non-receptor tyrosine kinase expressed in most hematopoietic cells, especially in B cells, myeloid cells, and platelets, with limited to undetectable levels in T lymphocytes and terminally differentiated plasma cells (Burger, 2019; Tasso et al., 2021). Activation of BTK results in the differentiation of antibody-producing plasma cells as well as B-cell survival and proliferation *via* inhibition of Fas/CD95-induced apoptosis in lymphoid B cells. BTK is involved in a number of signaling pathways including toll-like receptor (TLR), Fc Receptor (FcR), and B-cell receptor (BCR) signaling pathways (Alu et al., 2022), as well as several downstream signaling cascades such as the NF-κB, PI3K, and MAPK pathways (Burger, 2019; Liang et al., 2018). BTK is critical for the secretion of pro-inflammatory cytokines (Tasso et al., 2021). Antagonism of BTK leads to the inhibition of BCR-dependent B-cell activation and proliferation and inhibits the production of inflammatory cytokines in innate immune cells (Alu et al., 2022). Therefore, BTK over-activation is postulated to contribute to chronic inflammation, and BTK inhibition is a therapeutic target for B-cell malignancies and autoimmune diseases (Tasso et al., 2021; Alu et al., 2022). Mutations in BTK are the cause of X-linked agammaglobulinemia (XLA) in humans, which is an inherited immune disorder caused by an inability to produce B cells or B-cell-derived immunoglobulins. People with XLA have extremely low numbers of B cells and low levels of all types of immunoglobulins. They also fail to develop antibodies to specific antigens and will not produce protective antibodies after immunizations (Winkelstein et al., 2006). BTK knockout mice exhibit a phenotype that partially resembles human XLA where they have a reduction in splenic and peripheral B-cells and are unable to mount a thymus-independent type II response to antigens (Ellmeier et al., 2000). BTK inhibitors have also been observed to reduce inflammation and the production of autoantibodies (Alu et al., 2022).

The BTK inhibitors acalabrutinib (CALQUENCE®), ibrutinib (IMBRUVICA®), pirtobrutinib (JAYPIRCA®), and zanubrutinib (BRUKINSA®) are currently approved for oncology indications, such as lymphomas and B-cell malignancies, and are currently being investigated for additional therapeutic indications such as multiple sclerosis (MS), rheumatoid arthritis (RA), and other immune-mediated diseases (Tasso et al., 2021; Akinleye et al., 2013). BTK inhibitors can be either reversible (non-covalent) or irreversible (covalent) binders to BTK. Acalabrutinib, ibrutinib, and zanubrutinib are irreversible, covalent binders to BTK. Irreversible, covalent binding is cited as enhancing the therapeutic effect (Alu et al., 2022), while reversible (non-covalent) binders may produce reduced side effects compared to irreversible counterparts (Tasso et al., 2021).

##### 3.3.2.1 Overview of key health hazards

Given their MOA, the critical effect of concern for unintended exposure to BTK inhibitors is an impact on innate and humoral immunity. Inhibition of BTK results in B cells not reaching maturity and therefore less plasma cells and antibodies being produced. Therefore, a lack of B-cell proliferation and differentiation is anticipated to increase susceptibility to viral and/or bacterial infection or cause reduced vaccination efficacy (Alu et al., 2022). Aligned with this, incidences of infection have been cited as being characteristic upon treatment with ibrutinib (Burger, 2019). Additionally, invasive fungal infections have emerged as a safety concern in patients treated with BTK inhibitors with the hypothesized MOA being the inhibition of macrophages suppressing phagocytosis of aspergillus (Burger, 2019). Cardiac arrhythmias have also been noted as potential adverse reactions related to BTK inhibitor treatment, however it is unclear whether such adverse reactions are due to off-target effects that may be unlikely with second-generation BTK inhibitors (Burger, 2019).

##### 3.3.2.2 Mutagenicity and carcinogenicity

None of the BTK inhibitors are known to be mutagenic or clastogenic, and carcinogenicity studies were either lacking or negative (Table 1). The impact of BTK inhibition on the development of malignancies (i.e., tumorigenic potential) is not known; however, secondary malignancies (especially non-melanoma skin cancers) have been observed in clinical studies and are considered to be a class risk, with advice to use sun protection and monitor for new cancers. More broadly, malignancies have been identified as a potential concern for immunomodulatory agents. Malignancies have been reported in patients with XLA, including lymphoreticular malignancies, gastric and colorectal adenocarcinoma, and squamous cell carcinoma of the lung (Winkelstein et al., 2006).

##### 3.3.2.3 Developmental and reproductive toxicity

Developmental toxicity is anticipated to be a potential health hazard based on available information (AstraZeneca Pharmaceuticals, 2024; Pharmacyclics LLC, 2024; Eli Lilly and Company, 2024; BeiGene USA, 2024). Of the four FDA-approved BTK inhibitors, all were negative for reproductive toxicity, and all were positive for developmental toxicity (Table 1). No adverse effects on fertility were noted for the BTK inhibitors based on their respective product labels.

##### 3.3.2.4 Potential off-target effects

Ibrutinib, a first-generation BTK inhibitor, has been noted to have the potential for off-target toxicities as it can inhibit other kinases that harbor a homologous cysteine in the ATP-binding site—including EGFR/ERBB-family (EGFR, HER2/ERBB2, HER4/ERBB4) and TEC-family kinases (ITK, TEC, BMX, RLK [TXK]). It can covalently engage EGFR at Cys797, albeit less efficiently than purpose-built EGFR inhibitors. In T cells, functional ITK inhibition has been shown to skew immune responses toward a Th1 phenotype (Stankovic and Kwok, 2021). Acalabrutinib is a covalent, second generation BTK inhibitor that has higher selectivity (does not inhibit EGFR signaling, ITK, or TEC kinases compared to ibrutinib), while zanibrutinib is another covalent BTK inhibitor that is more selective than ibrutinib as well (Guo et al., 2019).

##### 3.3.2.5 Sensitive subpopulations

Given the MOA, immunocompromised individuals are anticipated to be a sensitive subpopulation for unintended exposure to BTK inhibitors in the workplace. Further, all FDA-approved BTK inhibitors have reduced dosing recommendations in patients with renal and/or hepatic impairment.

##### 3.3.2.6 Other concerns and considerations

None of the FDA-approved BTK inhibitors have black box warnings. With regards to translational effects, BTK inhibitors are known to cause histopathologic effects in the pancreas specifically in rats, and this is considered a class effect not relevant to safety assessment in humans.

##### 3.3.2.7 Hazard classifications for safety data sheets

Based on health hazard data for the four FDA-approved BTK inhibitors, a GHS classification for reproductive toxicity (developmental) may be warranted for BTK inhibitors lacking data for this endpoint.

##### 3.3.2.8 Bringing it all together—considerations for estimating OELs for BTK inhibitors

The MOA of inhibiting BTK, a key enzyme in B-cell receptor signaling, is effective against malignant B cells, but can also affect normal B-cell function and lead to immunosuppression. The potential for BTK inhibitors to act as reversible (non-covalent) or irreversible (covalent) binders to BTK influences their duration of action, potency, and off-target effects, with covalent binders typically leading to more sustained inhibition, and potential toxicity, following occasional exposures. Further, the resynthesis rate of BTK in human whole blood has been reported to be 115–154 h (Sung et al., 2021). In another report, the half-life of BTK was measured to be 48–72 h in purified human B cells (Sung et al., 2021). Additionally, patient-specific factors such as age, organ function (especially liver and kidneys), and genetic polymorphisms (e.g., CYP enzyme variations) can influence drug metabolism, clearance, and toxicity. The latter is especially relevant for BTK inhibitors as many undergo hepatic metabolism *via* the CYP3A4 pathway. Given the narrow therapeutic indices of BTK inhibitors, low-dose extrapolations could be warranted since BTK inhibitors may have immunomodulatory activity at subtherapeutic exposures. BTK inhibitors can potentially interfere with B-cell function, and unintended exposure can pose risks to immune system development during pregnancy. First-generation BTK inhibitors have the potential to inhibit other kinases, namely EGFR/ERBB- and TEC-family kinases; however, second-generation BTK inhibitors have demonstrated greater selectivity and thus lower potential for off-target effects than their predecessors. In light of these factors, OELs were estimated for orally administered FDA-approved BTK inhibitors (*n* = 4) using the therapeutic dose divided by the safety factor approach as described in Section 2.4 and in Faria et al. and Ku (Faria et al., 2016; Ku, 2000). The estimated OELs for BTK inhibitors range from 5 to 14 μg/m^3^ (Figure 4; Supplementary Table S2) spanning OEB 2 and 3A, (Figure 3).

#### 3.3.3 CDK inhibitors

Cyclin-dependent kinases (CDKs) are a multifunctional family of enzymes capable of modifying various protein substrates involved in cell cycle development. CDKs are serine/threonine kinases, and their activity is dependent on the “cyclin” regulatory subunit. Further, CDKs interact with one another at different checkpoints of the cell cycle including progression between the S and M phases to ensure that each cell accurately replicates its DNA and that equal segregation occurs between two daughter cells. To date, twenty CDKs and 29 cyclins have been identified in human cells that are known to regulate the cell cycle, gene transcription, and RNA splicing. CDK-1, -2, -3, -4, and -6 are believed to directly regulate cell cycle division and transition, while CDK-7, -8, -9, -12, −13 are thought to play a role in gene transcription (Canavese et al., 2012; Fisher, 2012; Łukasik et al., 2021; Malumbres, 2014; Cic et al., 2015). Dysregulation of CDKs can impact cell cycle stages and gene transcription leading to apoptosis. If dysregulation is not halted or corrected, it can lead to neurodegenerative diseases (Alzheimer’s, Parkinson’s), stroke, and cancer (Łukasik et al., 2021; Malumbres, 2014).

CDK inhibitors have been under consideration since the 1990s, and the first generation includes pan-CDK inhibitors such as flavopiridol and roscovitine. However, due to poor selectivity and significant safety and toxicity concerns, these inhibitors failed in the clinic. Since then, approximately 40 second-generation pan-CDK inhibitors with improved selectivity and toxicology profiles have been developed and are currently in various stages of development (Zhang et al., 2021).

The FDA approved CDK inhibitors abemaciclib (VERZENIO®), palbociclib (IBRANCE®), ribociclib (KISQALI®), and trilaciclib (COSELA®) primarily inhibit CDK4/6 activity and are being used either as a monotherapy or combination therapy for the treatment of breast cancer and prevention of chemotherapy-induced myelosuppression. Abemaciclib, palbociclib, and ribociclib are capable of arresting cell cycle from G1 to S and blocking phosphorylation of Rb protein leading to inhibition of proliferation of Rb-positive tumor cells (Zhang et al., 2021; Eli Lilly and Company, 2023; Pfizer, 2023a; Novartis Pharmaceuticals Corporation, 2023). Trilaciclib is approved for treatment to reduce the incidence of chemotherapy-induced myelosuppression in adult patients with small cell lung cancer (G1 Therapeutics, 2023).

##### 3.3.3.1 Overview of key health hazards

Although development and application of CDK inhibitors show great promise and progress for the treatment of various diseases, including cancer, there are significant toxicity concerns associated with these inhibitors. For example, first generation pan-CDK inhibitors exhibited high toxicity that caused unintended effects in normal cells (Zhang et al., 2021; Meijer et al., 1997; Whittaker et al., 2017). Three of the four FDA-approved CDK4/6 inhibitors abemaciclib, palbociclib, and ribociclib exhibited similar side effects. The most common grade 3/4 adverse reactions include neutropenia, leukopenia, and diarrhea. Other adverse reactions of any grade include thrombocytopenia, anemia, lymphopenia, fatigue, nausea, arthralgia, alopecia, infections, anemia, vomiting, abdominal pain, and decreased appetite (T et al., 2018; Yang et al., 2021). Additionally, there is a risk of interstitial lung disease/pneumonitis, hepatotoxicity, venous thromboembolism, and embryo fetal toxicity (Eli Lilly and Company, 2023; Pfizer, 2023a; Novartis Pharmaceuticals Corporation, 2023).

##### 3.3.3.2 Mutagenicity and carcinogenicity

None of the FDA-approved CDK inhibitors are known to be mutagenic or clastogenic; however, palbociclib was aneugenic *in vitro* and *in vivo*. Carcinogenicity studies were either lacking or negative, apart from palbociclib, which was positive in rats and negative in mice (Table 1) (Eli Lilly and Company, 2023; Pfizer, 2023a; Novartis Pharmaceuticals Corporation, 2023; G1 Therapeutics, 2023).

##### 3.3.3.3 Developmental and reproductive toxicity

All four FDA-approved CDK inhibitors exhibited potential for reproductive toxicity or developmental toxicity (Table 1). In reproductive toxicity studies with abemaciclib, palbociclib, and ribociclib, there was no effect on the reproductive function, fertility, or early embryonic development in female rats. However, there were drug-related findings observed in male rats and dogs in the testis, epididymis, prostate, and seminal vesicle. In embryo-fetal development studies, a decrease in fetal body weight and increased incidence of cardiovascular and skeletal malformations were reported in fetuses from pregnant rats and rabbits. Additionally, mortality resulting from severe anemia was observed in CDK4/6 double knockout mice in late stages of fetal development (Eli Lilly and Company, 2023; Pfizer, 2023a; Novartis Pharmaceuticals Corporation, 2023; G1 Therapeutics, 2023). Fertility studies have not yet been performed with trilaciclib. However, female rats and dogs treated with trilaciclib exhibited reductions in mean ovary and uterus weights at clinically relevant doses. These findings were reported to be reversible following cessation of exposure (G1 Therapeutics, 2023).

##### 3.3.3.4 Potential off-target effects

The investigated CDK inhibitor, flavopiridol, has been associated with various off-target effects such as cell cycle inhibition, transcriptional errors, autophagy, apoptosis, and endoplasmic reticulum stress. These effects are likely attributed to a lack of specificity given that CDK inhibitors 1) broadly target several protein kinases (e.g., CDK1 and CDK9) critical for cell proliferation and survival, respectively, and 2) do not accurately discriminate between healthy and cancerous cells. This lack of specificity also causes toxicity and adverse reactions that are widely observed with CDK inhibitors such as abemaciclib, palbociclib, ribociclib, and trilaciclib (Eli Lilly and Company, 2023; Pfizer, 2023a; Novartis Pharmaceuticals Corporation, 2023; G1 Therapeutics, 2023; Asghar et al., 2015).

##### 3.3.3.5 Sensitive subpopulations

Given the MOA, patients with pre-existing neutropenia or liver insufficiency are anticipated to be sensitive subpopulations for unintended exposure to CDK inhibitors in the workplace. Further, one marketed CDK inhibitor (abemaciclib) has a reduced dosing recommendations in patients with hepatic impairment.

##### 3.3.3.6 Other concerns and considerations

None of the marketed CDK inhibitors have black box warnings. With regards to translational effects, CDK inhibitors are known to cause neutropenia, leukopenia, and diarrhea. Additionally, there is a risk of interstitial lung disease/pneumonitis, hepatotoxicity, venous thromboembolism, and embryo fetal toxicity.

##### 3.3.3.7 Hazard classifications for safety data sheets

Based on health hazard data for the four FDA-approved CDK inhibitors, GHS classification for reproductive toxicity (developmental as well as male and female fertility) may be warranted for CDK inhibitors lacking data for these endpoints.

##### 3.3.3.8 Bringing it all together—considerations for estimating OELs for CDK inhibitors

The MOA of inhibiting CDKs, which regulate cell cycle progression, may lead to controlled cell division in cancer cells but potentially affect normal cells with high turnover rates. CDK inhibitors can potentially disrupt cell division during embryonic and fetal development, and unintended exposure to these inhibitors can pose risks such as impaired growth and birth defects; further, male reproductive toxicity may be of concern based on nonclinical studies. CDK inhibitors are intended to target specific CDKs, but may affect other kinases or signaling pathways, and these off-target effects can lead to organ-specific toxicities such as hepatotoxicity, cardiotoxicity, myelosuppression, and various adverse reactions based on the prescribing information for approved CDK4/6-targeting inhibitors. Considering these factors, OELs were estimated for orally administered FDA-approved CDK inhibitors (*n* = 3) using the therapeutic dose divided by the safety factor approach as described in Section 2.4 and in Faria et al. and Ku (Faria et al., 2016; Ku, 2000). The estimated OELs for CDK inhibitors range from 3.8 to 20 μg/m^3^ (Figure 4; Supplementary Table S2) spanning OEB 2 and 3A, (Figure 3).

#### 3.3.4 JAK inhibitors

Janus kinases (JAKs) are a family of non-receptor tyrosine kinases that play a major role in signal transduction through the Janus kinase-signal transducer and activator of transcription (JAK-STAT) signaling pathway (Morris et al., 2018). JAKs are bound to the intracellular region of cytokine receptors and once a dimer is activated, JAK transphosphorylation occurs and leads to the recruitment of one or more STATs that will subsequently translocate to the nucleus to modulate gene expression (Stark and Darnell, 2012). JAKs are involved in the signaling of more than 50 cytokines and growth factors that impact processes such as inflammation, hematopoiesis, immune regulation, and apoptosis (Morris et al., 2018). There are four members to the JAK family: JAK1, JAK2, JAK3, and TYK2. Activation of specific JAK combinations of each cytokine leads to different downstream effects (Spinelli et al., 2021).

JAK1, JAK2, and TYK2 are ubiquitously expressed while JAK3 is primarily expressed in hematopoietic cells (Yamaoka et al., 2004; Tortolani et al., 1995). JAK1 is involved in the signaling of multiple cytokine families including receptors using shared subunits γc or gp130, and interferons (IFNs) (Rodig et al., 1998). JAK2 participates in the signal transduction of the IL-3 receptor family, hormone-like cytokines such as erythropoietin (EPO), growth hormone (GH), prolactin (PRL), and thrombopoietin (TPO) (Morris et al., 2018). JAK3 is only known to be involved in signal transduction for cytokines of the γc family (Hu et al., 2021). TYK2 transmits signals from cytokines of the IL-12 family, gp130 family, IL-10 family, and Type I IFN (Tanaka et al., 2022).

Dysregulation of the JAK-STAT pathway is associated with autoimmune, hematologic, immunodeficiency-related disorders, and various types of cancer. Because of the extensive involvement of the JAK family in pro-inflammatory cytokine signaling, targeted small-molecule JAK inhibitors have been and are currently tested in the clinic. To date, 11 JAK inhibitors are approved by the FDA: abrocitinib (CIBINQO®), baricitinib (OLUMIANT®), deucravatinib (SOTYKTU®), deuruxolitinib (LEQSELVI®), fedratinib (INREBIC®), momelotinib (OJJAARA®), pacritinib (VONJO®), ritlecitinib (LITFULO®), ruxolitinib (JAKAFI®; OPZELURA®), tofacitinib (tasocitinib, XELJANZ®), and upadacitinib (RINVOQ®) (Pfizer, 2023b; Sun Pharma, 2024; Bristol-Myers Squibb Company, 2022; Incyte Corporation, 2023a; Pfizer, 2023c; GlaxoSmithKline, 2023; Eli Lilly and Company, 2022; Incyte Corporation, 2023b; AbbVie, 2024; Bristol-Myers Squibb Company, 2023; CTI Biopharma Corp, 2023; Pfizer, 2024).

Selectivity of the approved JAK inhibitors varies; first-generation molecules block multiple JAKs while selective inhibitors such as upadacitinib (JAK1) and deucravacitinib (TYK2) are also marketed (Tanaka et al., 2022; Schwartz et al., 2017). JAK inhibitors are approved and being researched for treatment of rheumatologic, dermatologic, gastrointestinal, and neoplastic indications like atopic dermatitis, arthritis, graft-versus-host-disease, myelofibrosis, and polycythemia vera (Spinelli et al., 2021). They are all administered orally except for ruxolitinib which is available as a topical cream in addition to oral tablets (Pfizer, 2023b; Sun Pharma, 2024; Bristol-Myers Squibb Company, 2022; Incyte Corporation, 2023a; Pfizer, 2023c; GlaxoSmithKline, 2023; Eli Lilly and Company, 2022; Incyte Corporation, 2023b; AbbVie, 2024; Bristol-Myers Squibb Company, 2023; CTI Biopharma Corp, 2023; Pfizer, 2024).

##### 3.3.4.1 Overview of key health hazards

Immunosuppression is an expected effect of JAK inhibitors since their targets are deeply involved in immune regulation. Increased risk of infections such as upper respiratory tract infections and urinary tract infections has been reported with JAK inhibitors (Nash et al., 2021). Although there is some overlap in pathways affected by the inhibition of each JAK isoform, the adverse reactions caused by pan-JAK inhibitors and selective JAK inhibitors may differ due to the difference in inhibited cytokine signaling (Tanaka et al., 2022). For example, JAK2 inhibition causes cytopenias due to the blockade of hematopoietic growth factors signaling like EPO (Schwartz et al., 2017). Changes in clinical chemistry parameters including blood cell counts, hemoglobin, liver transaminase, creatine kinase, cholesterol, and creatinine have been observed in patients treated with JAK inhibitors (Harigai, 2019; Clarke et al., 2021). Knockout of JAK3 in mice and JAK3 deficiency in humans results in severe combined immunodeficiency (SCID) and dysfunctional mature T and B lymphocytes (Nosaka et al., 1995; Roberts et al., 2004). Human TYK2 deficiencies lead to increased susceptibility to viral and bacterial pathogens, likely due to defects in the transduction of IFN-I, IL-12, and IL-23 signaling (Casanova et al., 2012).

##### 3.3.4.2 Mutagenicity and carcinogenicity

None of the approved JAK inhibitors are known to be mutagenic or clastogenic (Table 1). Carcinogenicity studies were generally negative with some positive results with unknown relevance to humans above the recommended therapeutic doses (on an AUC basis) (Pfizer, 2023b; Bristol-Myers Squibb Company, 2022; Incyte Corporation, 2023a; Pfizer, 2023c; GlaxoSmithKline, 2023; Eli Lilly and Company, 2022; Incyte Corporation, 2023b; AbbVie, 2024; Bristol-Myers Squibb Company, 2023; CTI Biopharma Corp, 2023; Pfizer, 2024). Although not observed with JAK inhibitors in randomized clinical trials for rheumatoid arthritis, in the ORAL Surveillance tofacitinib study, the incidence rate for malignancy (i.e., incidence rate of lung cancer, lymphoma, and non-melanoma skin cancer) was increased compared to those treated with TNF inhibitors, however the extent of the increased risk is still unclear (Ytterberg et al., 2022; Winthrop and Cohen, 2022). In addition, there is conflicting evidence on a potential increased risk of developing lymphomas in patients with myeloproliferative neoplasms treated with JAK inhibitors (Nocturne et al., 2020; Rumi and Zibellini, 2019). The mechanism leading to a potential increased cancer risk is presently unknown.

##### 3.3.4.3 Developmental and reproductive toxicity

DART, including teratogenicity, was observed in nonclinical studies with JAK inhibitors (Table 1). A single exception, the TYK2 inhibitor deucravacitinib, did not cause effects on embryo-fetal development in animal studies (Pfizer, 2023b; Bristol-Myers Squibb Company, 2022; Incyte Corporation, 2023a; Pfizer, 2023c; GlaxoSmithKline, 2023; Eli Lilly and Company, 2022; Incyte Corporation, 2023b; AbbVie, 2024; Bristol-Myers Squibb Company, 2023; CTI Biopharma Corp, 2023; Pfizer, 2024). Evaluating the mechanistic relevance of these targets with respect to DART in animal models is challenging as JAK1 knockout mice die perinatally and exhibit neurological disease and severe lymphocyte damage (Rodig et al., 1998), and JAK2 knockout leads to embryonic lethality in mice due to impaired hematopoiesis (Neubauer et al., 1998).

##### 3.3.4.4 Sensitive subpopulations

Given the immunosuppressive effects of JAK inhibitors, patients and workers that are already immunocompromised can be considered a sensitive subpopulation. Results of the post-marketing ORAL Surveillance study have shown that rheumatoid arthritis patients are at increased risk of various serious adverse reactions, making them a sensitive subpopulation. Several of the currently marketed JAK inhibitors have reduced dosing recommendations or are not recommended for patients with renal and/or hepatic impairment.

##### 3.3.4.5 Other concerns and considerations

JAK inhibitors are intended to target multiple or specific JAK isoforms, but may affect other kinases or signaling pathways; however, the black box warnings are issued primarily for their on-target rather than off-target effects which can lead to serious infections, higher rates of all-cause mortality, malignancies, cardiovascular events, and thrombosis based on the prescribing information for approved JAK inhibitors. Following the ORAL Surveillance tofacitinib study in rheumatoid arthritis patients, black box warnings have been added to JAK inhibitors indicated for the treatment of inflammatory conditions (Kragstrup et al., 2022). Specifically, increased risk of serious infections, higher rate of all-cause mortality, higher rate of malignancies, higher rate of cardiovascular events, and increased incidence of thrombosis are warnings common to the class (Ytterberg et al., 2022; Kragstrup et al., 2022).

##### 3.3.4.6 Hazard classifications for safety data sheets

Based on health hazard data for the 11 FDA-approved JAK inhibitors, GHS classification for reproductive toxicity (developmental as well as male and female fertility) may be warranted for JAK inhibitors lacking data for these endpoints.

##### 3.3.4.7 Bringing it all together—considerations for estimating OELs for JAK inhibitors

The MOA of inhibiting JAKs, which regulate signaling for cytokines and growth factors, suppresses immune responses, is beneficial for autoimmune conditions like rheumatoid arthritis, but increases the risk of infections and malignancies due to immunosuppression. Target presence should also be considered, as JAK1, JAK2, and TYK2 are ubiquitous, while JAK3 is mainly expressed in hematopoietic cells. JAK inhibitors can potentially disrupt JAK-STAT signaling during embryonic and fetal development, and unintended exposure can pose risks to the developing immune and hematological systems; further, male reproductive toxicity may be of concern based on nonclinical studies. Given these considerations, OELs were estimated for orally administered FDA-approved JAK inhibitors (*n* = 11) using the therapeutic dose divided by the safety factor approach as described in Section 2.4 and in Faria et al. and Ku (Faria et al., 2016; Ku, 2000). The estimated OELs for CDK inhibitors range from 0.1 to 20 μg/m^3^ (Figure 4; Supplementary Table S2) spanning OEB 2 and 3B, (Figure 3).

## 4 Discussion

### 4.1 Overall considerations in the safety assessment of SMKIs to guide estimation of OELs

This analysis aims to inform occupational toxicologists and safety professionals of potential human health hazards of SMKIs to establish OELs and OEBs for data-limited R&D settings. SMKIs can be quite potent, causing specific biological effects at low doses, requiring careful handling by workers on that account. While some of these toxicities might not be of concern at sub-therapeutic exposures in working populations, they must be considered when establishing OELs.

It is important to consider all available pharmacologic, pharmacokinetic, pharmacodynamic, and safety information from human experience if available when establishing an OEL for an SMKI, since nonclinical data may result in overly conservative values. Adverse reactions associated with SMKIs are often like traditional chemotherapies (e.g., bone marrow, gastrointestinal, reproductive tract toxicity), however the mechanisms are distinct, threshold-mediated, and usually related to on- or off-target kinase inhibition. The estimation of OELs is largely driven by the pharmacological potency, which varies depending on the indication and characteristics of each molecule or class. In this analysis, we used the minimum daily therapeutic dose as a surrogate LOAEL to ensure protection against unintended pharmacological activity, which is regarded as an adverse effect in occupational settings where no therapeutic benefit is expected. This approach is intentionally protective and may underestimate the true NOAEL for many SMKIs, contributing to the clustering of estimated OELs mapped to OEB 2-3, which is an appropriate range for data-limited SMKIs in early development.

Establishing OELs for SMKIs has broad implications for occupational toxicologists and safety professionals. SMKIs are increasingly being used in various therapeutic areas, especially oncology, which necessitates rigorous implementation of appropriate safety standards and handling practices for workers. A deep understanding of their pharmacological and toxicological properties directly informs these practices, but relevant health-hazard data are often limited in early development, necessitating adaptable risk-assessment approaches to bridge evidence gaps. Complementing these data-driven decisions, a harmonized hazard framework is essential. GHS classifications inform hazard identification, risk assessment, control measures, and the development of OELs, which in turn establish safe exposure limits to support transportation and worker safety.

### 4.2 MOA as a determinant of hazards, OELs, and OEBs by target family

The MOA is an important consideration for establishing OELs to prevent adverse effects associated with SMKIs. SMKIs have many targets, and those with oncology indications often target signaling pathways that involve TKs and contribute to the proliferation of tumor cells. TKs play critical roles in cell growth, differentiation, and survival, and thus sensitive target organs in animals or humans include those with actively proliferating cell populations, such as bone marrow, skin, as well as the gastrointestinal and reproductive tracts (Roskoski, 2025). A notable exception is the B-Raf proto-oncogene, serine/threonine kinase (BRAF) inhibitors, which are relatively selective for a mutated form of BRAF that is present in certain tumors but generally absent in normal tissues (Pratilas et al., 2012; Cheng et al., 2018).

Although the toxicity profiles of some SMKIs may appear like those of traditional “anti-mitotic” cancer therapies (e.g., carboplatin), the indirect acting nature of SMKIs means that such effects are presumed to occur *via* threshold-based mechanisms. Consequently, risk assessment paradigms or additional exposure controls (e.g., dedicated manufacturing equipment) that are sometimes applied to direct-acting anti-cancer therapeutics are typically not applicable when evaluating the risk of exposure to SMKIs outside of therapeutic exposure scenarios. Understanding the MOA(s) of SMKIs can enable occupational toxicologists to derive OELs that are anticipated to be protective against specific on-target effects in unintentionally exposed workers.

Additionally, many SMKIs have a narrow therapeutic index, underscoring the importance of minimizing unintentional exposures, as prolonged or repeated exposure can lead to cumulative off-target effects. In some cases, low-dose extrapolations may be warranted for SMKIs where biological activity has been observed at the lower bounds of pharmacological relevance. The clustering effects observed in this analysis can be attributed to 1) developmental toxicity as a class effect of SMKIs as well as 2) conservative assumptions (e.g., use of the minimum daily therapeutic dose as a surrogate LOAEL, high uncertainty factors), which compressed the estimated OELs into intermediate bands. Therefore, the absence of OEB one reflects developmental hazards being attributed to the MOA of SMKIs, and the absence of OEB 4 reflects their indirect, threshold-mediated MOAs rather than extreme cytotoxicity.

Differences in pharmacological selectivity and target tissue expression further contribute to variability in estimated OELs observed both across and within target families. SMKIs that exhibit broader kinase inhibition profiles or target kinases with ubiquitous expression across tissues (e.g., JAK or PI3K family members) are more likely to affect multiple physiological systems, resulting in narrower margins between pharmacological activity and adverse effects. In contrast, SMKIs with greater target selectivity or preferential activity against disease-associated or mutation-specific targets (e.g., BRAFV600-selective inhibitors) may demonstrate a wider separation between therapeutic and off-target effects, supporting less restrictive OEBs compared to JAK inhibitors. Within individual target families, variability in OELs may also reflect differences in kinase selectivity, binding mode (e.g., reversible *versus* irreversible inhibition), and tissue distribution of the primary target. For example, inhibitors targeting kinases predominantly expressed in immune or hematopoietic tissues may be associated with greater concern for immunological or hematological effects, whereas inhibitors targeting kinases with more restricted tissue expression may present lower hazard potential to worker health and safety in R&D settings at equivalent levels of target engagement. This is supported by the observation that all three BRAFV600-selective inhibitors evaluated in this analysis were mapped to OEB 2. These mechanistic considerations provide a biological basis for the observed heterogeneity in exposure banding and underscore the importance of incorporating pharmacological selectivity and target biology when interpreting OEL variability within target families. However, a deeper analysis of interfamily OEL variability is beyond the scope of this work, which focuses on default and family-level banding considerations.

For certain SMKI families, particularly BTK and JAK inhibitors, immunosuppression represents a primary on-target pharmacological effect and therefore warrants specific consideration in occupational risk assessment. Although these agents are designed to modulate immune signaling pathways, available evidence indicates that immunomodulatory effects are threshold-dependent and require sustained target engagement at exposures approaching therapeutic levels. Therefore, at occupational exposure levels anticipated to be well below those associated with pharmacological activity, meaningful immunosuppression is not expected. And while immunosuppression is considered to be a critical effect for BTK and JAK inhibitors, the application of standard adjustment factors to prevent target engagement is anticipated to provide adequate protection without the need for additional adjustment factors. This approach is consistent with the treatment of other on-target, threshold-mediated effects in OEL derivation and avoids introducing unnecessary conservatism in the absence of evidence for low-dose biological activity relevant to occupational exposures in R&D settings.

### 4.3 Considerations of genotoxic, developmental, and reproductive hazards in occupational risk assessment

In contrast to direct-acting anticancer therapeutics that interact with DNA, the intended MOA of SMKIs does not typically involve modification of individual nucleic acids. The clastogenic and aneugenic effects of SMKIs are typically regarded as secondary to kinase inhibition and are generally regarded as indirect threshold-mediated effects that are not anticipated to pose a risk of carcinogenicity at low doses (ICH M7). Unlike genotoxicity, developmental toxicity is expected to be associated with pharmacological activity (e.g., on-target effects). In the absence of anticipated off-target effects or mutagenicity, one can reasonably assume that the developmental hazard is attributed to the MOA. Similarly, if the reproductive hazard is attributed to the MOA, it would not be anticipated to occur at a dose where pharmacological activity is not expected. Essentially, DART hazards predicted to be associated with exposures at therapeutic doses would not be anticipated at doses where there is a lack of pharmacology (i.e., when parameters are within background levels).

### 4.4 Consideration of sensitive subpopulations in occupational risk assessment

While DART hazards associated with SMKIs are not expected to occur in the absence of pharmacological activity, interindividual variability in systemic exposure and target engagement remains an important consideration when evaluating potential risks in exposed sensitive subpopulations. Many individual-specific factors such as age, organ function (especially liver and kidneys), and genetic polymorphisms (e.g., CYP enzyme variations) were identified as having the potential to influence drug metabolism, clearance, and toxicity of the SMKIs evaluated. Patient populations identified as potential sensitive subpopulations may not be reflective of those that would be identified in the workplace as patient exposures are anticipated to be significantly higher (e.g., intentional parenteral injection vs. passive inhalation). Therefore, in the context of controlled workplace exposures, an adjustment for sensitive subpopulations may not be necessary. For example, individuals with severe hepatic and/or renal impairment would not be presumed to be part of the workforce. However, a healthy working population may include those with mild to moderate hepatic and/or renal impairment, so consideration may be warranted if the OEL is near a dose that may cause a biologically relevant effect in compromised individuals.

### 4.5 Implications for proposed default OEB and lifecycle management

Several frameworks have been proposed to guide OEL establishment for anticancer molecules, small molecule drug candidates, and novel pharmaceutical modalities, many of which have been informed by toxicological data on SMKIs. Based on the analysis presented herein, OEB 3A, reflecting an airborne concentration range of 1 to <10 μg/m^3^, represents a reasonable conservative default OEB assignment for SMKIs in data-limited R&D settings and DART or genotoxicity should be anticipated. In this analysis, we conservatively assumed a lower inhalation bioavailability would result in higher OELs, but this assumption was intentionally made to account for uncertainty in early development.

The proposed default OEB is intended for SMKIs in data-limited R&D settings, and in cases where the MOA and primary target(s) are known. In such cases, the default OEB provides a conservative, MOA-informed starting point to guide handling practices and control strategies until additional nonclinical or clinical data become available. Importantly, this default OEB is not intended to replace molecule-specific OELs. The default OEB should not be applied for molecules with demonstrated off-target or non-threshold toxicities, for molecules with robust nonclinical and clinical data, or in cases where refined OELs have already been established using molecule-specific pharmacokinetic and toxicological data. In these scenarios, reliance on a generic default band may result in either unnecessary conservatism or insufficient specificity. The proposed default OEB aligns with existing industry guidance and published frameworks for managing data-limited molecules including approaches described by Graham et al. and Glogovac et al., which similarly recommend OELs in the range of 1 to ≤10 μg/m^3^ for potent small-molecule APIs (Graham et al., 2020; Glogovac et al., 2021).

Consistent with these frameworks, the default OEB is intended to be revisited and refined as development progresses and additional health hazard data become available as well as any reported occupational effects. While they are useful for providing guidance in data-limited scenarios, OEBs and analogous banding systems are speculative OELs, and additional nonclinical and clinical data are needed to derive refined OELs, which allow for greater specificity, regulatory compliance, and precision in risk assessment. Moreover, to enhance the precision of toxicity predictions and mitigate remaining uncertainties, advanced risk assessment methods, such as physiologically based pharmacokinetic modelling (PBPK) and *in vitro*-to-*in vivo* extrapolation (IVIVE), can be used to predict potential liabilities in humans and refine HBELs to provide greater confidence that they are protective of worker health and safety in R&D settings.

It is also imperative to implement training programs for workers handling SMKIs in data-limited R&D settings. Training should cover the necessary engineering controls as well as the proper personal protective equipment, handling practices, and emergency response practices. Synergistically with the above approaches, OELs play a critical role in protecting worker health and safety for pharmaceuticals, including SMKIs, in R&D, manufacturing, and clinical exposure settings. Overall, establishing OELs for SMKIs necessitates a multifaceted approach that develops robust considerations based on toxicological data, incorporates advanced risk assessment methods, and implements comprehensive training programs, all while adhering to industry and regulatory guidance and working collaboratively to aim for global harmonization. Together, these coordinated efforts will help advance this important objective while safeguarding worker health and safety.

## 5 Conclusion

SMKIs are a prominent and growing class of approved drugs with diverse targets across therapeutic indications. Early development activities require occupational toxicologists to understand the health hazards presented by this class to establish protective OEBs for SMKIs lacking toxicological and pharmacological data. Based on the health hazard data for FDA-approved SMKIs, an airborne concentration range of one to ≤10 μg/m^3^ is anticipated to be protective of adverse pharmacological and toxicological effects in workers handling SMKIs based on an 8-h workday. Notably, some target families that may warrant more protective OEBs depending on their pharmacological potency include FGFR, JAK, MEK, mTOR, PI3K, TK, VEGFR, and VEGFR-TK inhibitors. As is the case with all HBELs, especially in data-limited R&D settings, OEBs and OELs should be re-evaluated throughout the molecules’ development and lifecycle to re-assess the appropriateness of the limits.

## Data Availability

The original contributions presented in the study are included in the article/Supplementary Material, further inquiries can be directed to the corresponding author.
